# From Colloidal Dispersions of Zeolite Monolayers to Effective Solid Catalysts in Transformations of Bulky Organic Molecules: Role of Freeze-Drying and Dialysis

**DOI:** 10.3390/molecules26072076

**Published:** 2021-04-04

**Authors:** Katarzyna Kałahurska, Pawel P. Ziemiański, Wieslaw J. Roth, Barbara Gil

**Affiliations:** 1 Faculty of Chemistry, Jagiellonian University, Gronostajowa 2, 30-387 Kraków, Poland; katarzyna.kalahurska@doctoral.uj.edu.pl (K.K.); wieslaw.roth@uj.edu.pl (W.J.R.); 2 Institute of Geological Sciences, Polish Academy of Sciences, Senacka 1, 31-002 Kraków, Poland; p.ziemianski@ingpan.krakow.pl

**Keywords:** 2D zeolites, colloidal suspensions, freeze-drying, lyophilization, dialysis

## Abstract

We investigated the properties and catalytic activity of zeolites with MWW topology obtained by unprecedented liquid exfoliation of the MCM-56 zeolite into solutions of monolayers and isolation/reassembly of the dispersed layers by various methods, with optional purification by dialysis or ammonium exchange. The layers were recovered by flocculation with alcohol or ammonium nitrate and freeze-drying. Flocculation alone, even with ammonium nitrate, did not ensure removal of residual sodium cations resulting in catalysts with low activity. Dialysis of the solutions with dispersed monolayers proved to be efficient in removing sodium cations and preserving microporosity. The monolayers were also isolated as solids by freeze-drying. The highest BET area and pore volume obtained with the freeze-dried sample confirmed lyophilization efficiency in preserving layer structure. The applied test reaction, Friedel–Crafts alkylation of mesitylene, showed high benzyl alcohol conversion due to increased concentration of accessible acid centers caused by the presence of secondary mesoporosity. The applied treatments did not change the acid strength of the external acid sites, which are the most important ones for converting bulky organic molecules. Zeolite acidity was not degraded in the course of exfoliation into monolayers, showing the potential of such colloid dispersions for the formation of active catalysts.

## 1. Introduction

Catalytic reactions take place at the active centers located at the available surfaces of a solid material. Their accessibility depends on the size of the reactant molecule on one hand, and on the number, nature and distribution of the active centers in a given catalyst, on the other. In the case of zeolites, having micropores that are hardly available to more bulky reactants due to pore sizes below 1 nm, the increase of accessibility can be achieved by decreasing the size of zeolite crystals, the introduction of secondary porosity, or by a combination of both approaches [1,2,3,4,5,6]. The ultimate size reduction is represented by layered, two-dimensional (2D) zeolites [7,8,9,10,11]. The intrinsic acid centers of such tens-to-hundreds-of-nanometers-wide- and only a few-nanometer-thin-crystals are all located near the external surface and thus are well accessible to bulky reactants. Even more important, such natural “flattening” of crystals does not seem to penalize either the acid sites strength or the microporous structure; thus, the intrinsic high activity and shape-selectivity may be retained [12,13].

Layered zeolite MCM-56, a representative of the MWW family, is a two-dimensional (2D) delaminated material obtained by direct synthesis, lending itself to easy modification [14,15]. MCM-56 contains 2.5 nm thick layers, with internal hexagonal channels of 0.55 nm diameter and surface cavities, so-called “external cups”, which are 0.7 nm wide and deep [16]. MCM-56 is very acidic due to high Al concentration (Si/Al~10).

The advent of 2D zeolites blurred the line that separated sharply rigid 3D zeolitic structures from flexible layered materials exemplified by clay minerals [17]. Complete exfoliation into monolayers in solution, so obvious and easy for clay minerals and most other 2D solids [18], has so far been very difficult to achieve with zeolites. Zeolite delamination [19] or multistep procedures involving swelling, shearing in a melt, and purification [20,21] produced monolayer zeolite suspensions in low yields, which thwarted subsequent use in bulk preparations. Recently, this ultimate manifestation of the layered nature has been reported as the dispersion of MCM-56 into colloidal suspensions of monolayers by soft-chemical liquid exfoliation [22]. Such exfoliated layers are isotropically dispersed in a solvent [23], and each layer is like a gigantic flat molecule. Colloidal suspensions of zeolite are monolayer analogs of the well-known colloidal suspensions of nanoclays [24], i.e., layered silicates with two-dimensional platelets of nanoscale thickness and a length of tens of nanometers.

Colloidal suspensions of monolayers may be reassembled into hierarchical materials to produce, among others, catalysts. To obtain active heterogeneous catalysts, the colloids must be precipitated and, since they are built of inactive zeolite crystals containing the template, must be activated. The activation procedure includes removing the residual template and sodium cations balancing the negative network charge, the chemical agent used for exfoliation, and other possible contaminants.

Such postsynthesis treatments must be carefully designed to avoid deactivation and degradation during aggregation of the layers [4,5] as their active centers are in the open and may be vulnerable. In the present paper, we analyze the effect of various treatments and purification procedures, including precipitation by flocculation with alcohol and ammonium nitrate, freeze-drying, and purification/activation by dialysis, classic calcination and ion exchange procedure. Both freeze-drying and dialysis are environmentally friendly methods because no chemicals are used, and no additional waste is created. It must be stressed that to date, only one zeolite structure, MWW, described in this article, has been reported to form the fully exfoliated material by a soft-chemical treatment [22], and the present paper deals with the previously unexplored methodology of flocculation of layers from a solution and their activation to obtain effective solid catalysts.

## 2. Results and Discussion

**Scheme 1:**
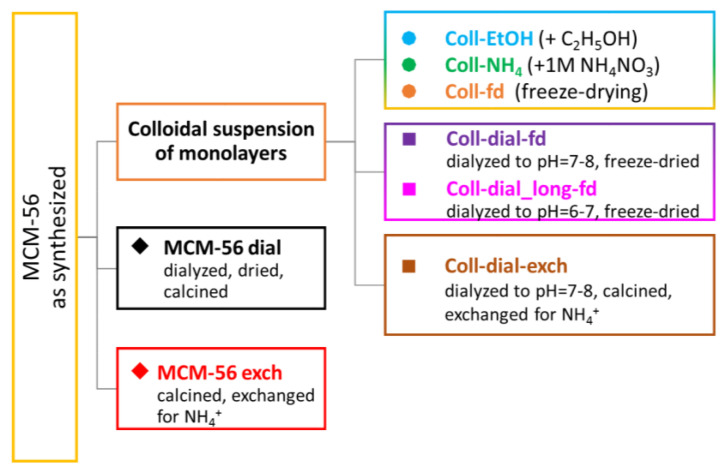
General outline of the treatments of the MWW colloidal dispersions and solid MCM-56. Samples in this scheme and all figures are coded by individual colors and symbols: diamonds (◆) for the solid MCM-56, circles (●) for precipitated samples, and squares (■) for dialyzed samples.

Colloidal dispersions of MCM-56 zeolite monolayers were produced by the treatment with 10–11.5% solutions of tetrabutylammonium hydroxide (TBAOH), which resulted in expansion, i.e., opening of interlayer galleries across original multilayered particles and producing total delamination/exfoliation of monolayers in solution (for detailed procedure see Section 3 and ref. [22]). The obtained colloidal suspensions of the MWW layers were precipitated by the addition of 95% ethanol (coll-EtOH) or 1 M NH_4_NO_3_ (coll-NH_4_) or isolated by freeze-drying (coll-fd). The general outline of the treatment scheme for obtained colloidal dispersions and sample coding is presented in Scheme 1.

All X-ray diffraction patterns (XRDs) indicate preservation of the MWW structure, which can be identified by intralayer reflections at ca 7, 14, 25 and 26° 2θ (Cu K_α_ radiation used throughout, λ = 0.154 nm), with Miller indices 100, 200, 220 and 310, respectively. In some cases (especially coll-dial_long-fd sample), the scattering intensity is lowered as a result of spatial disorder and deformation of layers rather than due to the formation of amorphous phase (amorphization could be due to increased pH during exfoliation).

**Figure 1:**
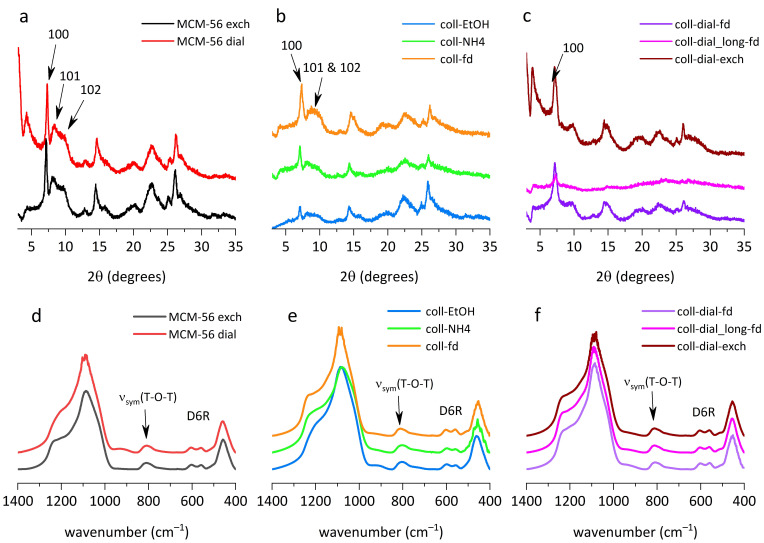
X-ray diffraction patterns and infrared (IR) spectra in KBr technique for the zeolites under study: (**a**,**d**) MCM-56 as synthesized and dialyzed, (**b**,**e**) colloids precipitated with ethanol, ammonium nitrate and freeze-drying (lyophilization), (**c**,**f**) colloids purified by dialysis and subsequently precipitated. All samples were additionally calcined before IR and XRD measurement.

The region 8–10° 2θ with the broad band characterizing MCM-56 is preserved in some samples, mostly without the appearance of a dip shown by the solid MCM-56 material (MCM-56 exch, Figure 1a). Such dip is considered to indicate some layer order; after forming the colloids and their subsequent precipitation, the layers appear to be less ordered than in the reference MCM-56 material. Precipitation by freeze-drying results in a sample (coll-fd, Figure 1b), which is less ordered than those precipitated with ethanol or ammonium nitrate. That confirms the positive effect of freeze-drying on the disorder of zeolite layers, both for colloids and the MCM-56 powder.

The solid materials isolated after exfoliation consist of randomly packed layers and are highly disordered systems with possible layer deformations. They cannot be well characterized based on XRD, allowing only some general qualitative conclusion and comparison to MCM-56 itself, which, being disordered, also provides limited information in its XRD. The crystallographic quality and preservation of the MWW structure of the layers upon treatment with TBAOH and exfoliation were confirmed by other means (e.g., transmission electron microscopy, TEM and atomic force microscopy, AFM) and presented in the original paper [22]. For these reasons, additional characterization presented below is required.

Preserved integrity of the layers was verified by quantitative evaluation of the infrared (IR) spectra in the pseudo-skeletal region obtained with the KBr technique in transmittance mode. Zeolite materials with the MWW framework show an IR double band at 500–600 cm^−1^ assigned to the δ_T-O-T_ vibrations (external bending of tetrahedra) of D6R (double six-membered ring) secondary building units. It was proven [25] that integrated intensity of this band correlates well with crystallinity, micropore volume and Brønsted acid sites (BAS) concentration and thus can be used for determining crystallinity of MCM-56 and quantitative determination of its content in a sample. For all samples, the integrated intensity of this band was practically the same (Figure 1d,e,f), which confirmed that the layers were not damaged despite various treatments.

**Figure 2:**
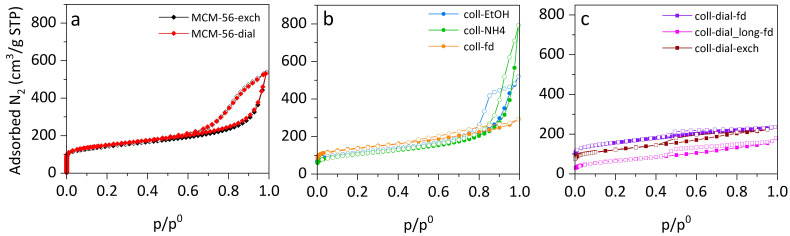
N_2_ adsorption–desorption isotherms for zeolites under study: (**a**) MCM-56 ion-exchanged and dialyzed, (**b**) colloids precipitated with ethanol, ammonium nitrate and freeze-dried (lyophilized), (**c**) colloids purified by dialysis and subsequently precipitated. All samples were additionally calcined before the isotherm measurements.

To further evaluate possible layer packing, the porosity of the samples after precipitation was investigated (Figure 2, Table 1).

**Figure 3:**
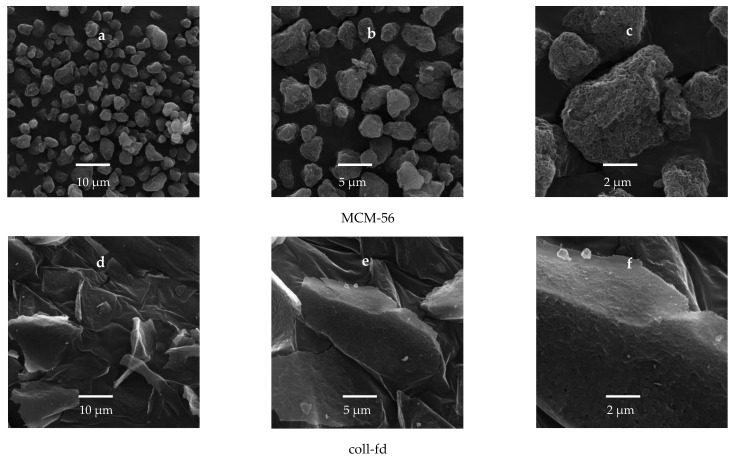

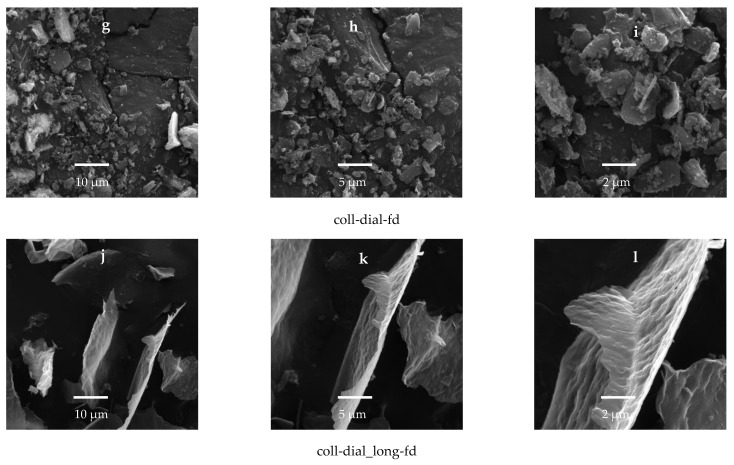
SEM images for representative zeolites: reference MCM-56 (**a**–**c**), coll-fd (**d**–**f**), coll-dial-fd (**g**–**i**), and coll‑dial_long-fd (**j**–**l**).

Isotherms for the reference MCM-56 zeolite are the same for the classically treated and dialyzed materials (Figure 2a), showing that dialysis does not change the layers’ arrangement in the solid product (specific surface area, S_BET_ and external surface area, S_ext_ are almost the same), also micropore volume is unchanged. For materials precipitated after exfoliation (Figure 2b), the isotherms are quite different at high p/p^0^ (>0.7) and are characteristic for wide intercrystalline mesopores and/or narrow macropores, dependent on the organization of stacks of the layers. The external surfaces are quite similar for all three samples (from 220 to 247 m^2^/g). The micropore volume is increasing, from 0.035 cm^3^/g for coll-NH_4_ to 0.086 cm^3^/g for freeze-dried sample (coll-fd). When the colloidal sample was dialyzed before lyophilization, the micropore volume was even higher (0.108 cm^3^/g, increase by 20%). This shows the advantage of isolation by lyophilization in preserving microporosity. The freeze-drying procedure caused a decrease in external surface (to 171 m^2^/g for coll-fd sample), which could be due to specific arrangement of the layers that can be observed in SEM images at high magnification (Figure 3) where packing of small, lamellar crystals resembles the arrangement of fish scales. Prolonged dialysis (sample coll-dial_long-fd) is not recommended because it has negative influence on the microporosity of samples–the BET area and V_micro_ values decrease (to 240 m^2^/g and 0.010 cm^3^/g), while the S_ext_ stays at the same level (175 m^2^/g).

It is important to mention that the freeze-dried sample is fluffy with very low-density. It is easy to handle and grind, while the other two samples are hard and difficult to separate.

The organization of the layers was observed by SEM for representative samples. The reference MCM-56 has the characteristic MWW material appearance, with plate-like crystals aggregated into bigger, rounded structures, with sizes in the range 2–5 μm (Figure 3a–c). Colloid dialysis (Figure 3c–e) results in altered layer arrangement with the formation of thin corrugated sheets of various sizes. At higher magnification, it can be seen that they are built of multiple small and thin crystals, packed like fish scales. When colloid dialysis is followed by isolation by freeze-drying, the sheets are probably broken into much smaller pieces, as seen in Figure 3g–i. The sample that was dialyzed for a long time again precipitated in the form of wide, thin sheets. Subsequent lyophilization of the already precipitated sample did not break up the sheets.

**Scheme 2:**
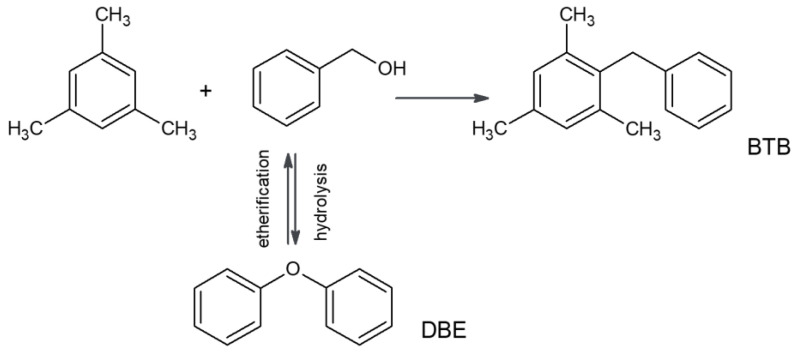
Alkylation of mesitylene with benzyl alcohol.

**Figure 4:**
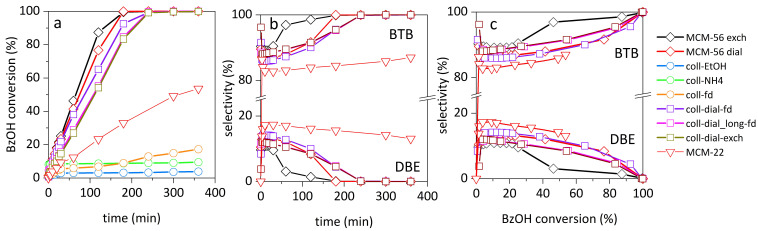
Catalytic test results. (**a**) selectivity of benzyl alcohol (BzOH) vs. time of reaction for all investigated catalysts, (**b**) selectivity to 2-benzyl-1,3,5-trimethylbenzene (BTB) and dibenzyl ether (DBE) vs. time, (**c**) selectivity to 2-benzyl-1,3,5-trimethylbenzene (BTB) and dibenzyl ether (DBE) vs. conversion of benzyl alcohol (BzOH).

The obtained materials were tested as acid catalysts for the transformation of bulky organic molecules. Friedel–Crafts alkylation of mesitylene with benzyl alcohol (BzOH) was chosen as a test reaction (Scheme 2, Figure 4, Table 1). This is a frequently selected test reaction because a clear trend is observed between the benzyl alcohol conversion and the presence of secondary mesoporosity [26] due to increased concentration of accessible acid centers [27].

With two possible reaction products, the main, bulkier one, 2-benzyl-1,3,5-trimethylbenzene (BTB), can be formed exclusively on the external surfaces of the zeolite crystals mainly because 1,3,5-trimethylbenzene (molecular dimensions 0.425 × 0.829 × 0.888 nm [28], kinetic diameter 0.818 nm [29]) cannot enter 10-membered rings, restricting access to the MCM-56 micropores (ca. 0.5 nm). The side product, dibenzyl ether (DBE), being the product of the condensation of two benzyl alcohol molecules, can be formed both at the external surfaces and inside micropores. For longer reaction times, the ether is transformed into the main product via hydrolysis to benzyl alcohol and subsequent alkylation [30].

The samples that were just precipitated (coll-EtOH, coll-NH_4_, coll-fd) were practically inactive, with benzyl alcohol conversions 5–20% (Figure 4a). Inactivity of the precipitated materials results from the presence of residual sodium cations (the present organics, i.e., hexamethyleneimine, HMI and residual tetrabutylammonium hydroxide, TBAOH, were removed before catalytic testing by high-temperature activation). This effect due to sodium cations will be discussed two paragraphs down.

Dialysis gave very active samples. All dialyzed materials reached 100% conversion of benzyl alcohol after a similar reaction time, with reaction half-time (t_1/2_) in the range 79–112 min. For comparison, t_1/2_ for the reference MCM-56 sample was 65 min, and for MCM-22 the longest of all, 167 min. Selectivities for all materials were very similar (Figure 4b,c), showing that the reaction mechanism was not changed, independently of the changes in the external surface or BAS and BAS_ext_ concentration. It is best seen in Figure 4c, where selectivity is presented as a function of conversion. Among all of the samples, the one purified by dialysis and then isolated by freeze-drying (coll-dial-fd) was the most active, with t_1/2_ 79 min. This means that zeolite acidity is not degraded during exfoliation into single monolayers, contrary to other types of delaminated zeolites obtained by swelling and precipitation by acidification. It shows the potential of the colloid dispersion of zeolite layers for the synthesis of active catalysts.

**Figure 5:**
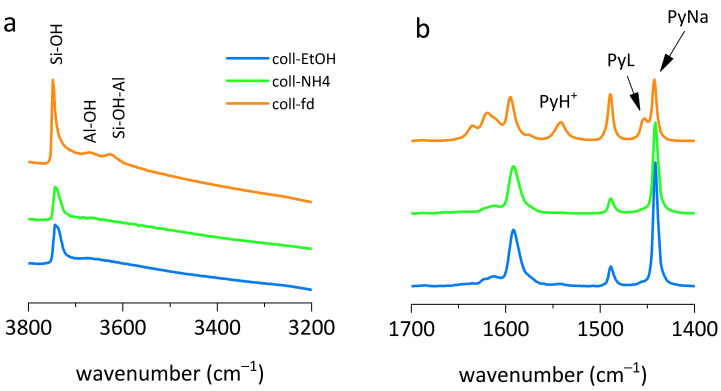
IR spectra of the calcined coll-EtOH, coll-NH_4_, and coll-fd samples in the OH vibrations region (**a**) and after pyridine adsorption (**b**).

As mentioned above, the layered material obtained by straight precipitation of colloids showed low activity, which is caused by the presence of residual amounts of sodium cations, neutralizing the negative charge of framework Al atoms. The sodium content could be determined by elemental analysis, but the accuracy of this method is not very high, especially when this content is low and the difference between the concentration in measured samples is at the level of experimental accuracy. Infrared (IR) spectroscopy can be used to determine the relative content of sodium cations by comparing the intensity of the 1442 cm^−1^ maximum of pyridine interacting with Na^+^. This maximum (Figure 5b) is the most intense in the coll-EtOH sample and the least intense in the freeze-dried sample. It is worth noting that even though the coll-fd sample has BAS concentration of 306 μmol/g, the maximum BzOH conversion is below 20%. Pyridine is probably small enough to fit into the zeolite channel next to the large sodium cation and can react with acid sites to form pyridinium cation, but the space is not large enough to accommodate larger reactant molecules.

The effect of dialysis on the properties of prepared colloids was examined and compared with the effect it had on the reference sample, the MCM-56 powder. Purification of colloids by dialysis was intended to remove the TBAOH used as the exfoliating agent as well as residual sodium cations. The purification effectiveness was traced by the change of pH of the colloidal solution, from initial pH = 12–13 to the final pH = 7–8, which usually happened after several hours of treatment (samples coll-dial-fd and coll-dial-exch). Further lowering of pH to the level 6–7 required several days and caused the colloid flocculation (sample coll-dial_long-fd).

Samples that were dialyzed before precipitation show a new feature in the XRD, i.e., a distinct broad peak near 10° 2θ (Figure 1c). Its origin is uncertain and difficult to rationalize, so it must be treated as a puzzle to be solved.

**Figure 6:**
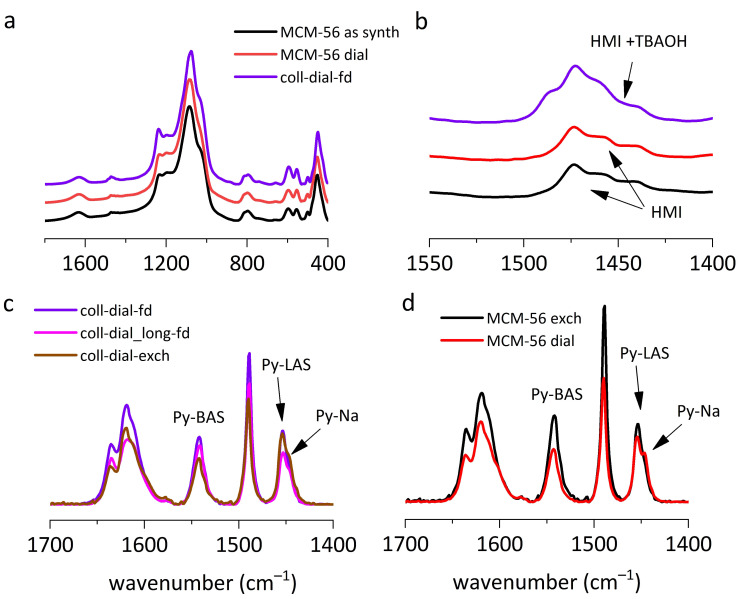
(**a**) Effect of dialysis on sodium, hexamethylene imine (HMI) and tetrabutylammonium hydroxide (TBAOH) content: IR spectra in KBr technique for dialyzed zeolites: MCM-56 as synthesized, MCM-56 dialyzed, and coll-dial-fd, (**b**) the spectra presented in (**a**) in the region of C-H and C-N vibrations characteristic of HMI and TBAOH, (**c**) effect of dialysis and ion exchange: IR spectra of pyridine adsorbed on colloids dialyzed for different times (coll-dial-fd and coll-dial_long-fd) and on sample ion exchanged after dialysis (coll-dial-exch), (**d**) effect of dialysis on reference MCM-56 zeolite: IR spectra of pyridine adsorbed on MCM-56 ion-exchanged and dialyzed samples.

IR spectra recorded in the KBr technique confirmed that dialysis did not change the template (HMI) content (Figure 6a,b), while the Na^+^ content (followed by pyridine sorption) decreased (Figure 6c,d). All dialyzed samples were catalytically active and had high BAS concentration (550–600 μmoL/g). The concentration of the BAS_ext_ was also high and constituted almost 30% of all BAS. It must be kept in mind that in MWW zeolites, BAS can be located both inside the channels accessible only through the 10-rings opening of ca. 0.5 nm in diameter, or in the surface pockets with an inner free-diameter of 0.71 nm and a depth of about 0.7 nm. The concentration of the external Brønsted acid sites was, therefore, determined by pivalonitrile adsorption, with a diameter of 0.62 nm [31,32]. Pivalonitrile probed only the acid centers located at the surface pockets and at the pore-mouth of 10-rings channels; such sites are referred to as BAS_ext_. When a dialyzed sample was additionally calcined and exchanged with NH_4_^+^ cations (sample coll-dial-exch), all parameters (BET surface, BAS and BAS_ext_ concentration) were lower than for dialyzed material. This translated into catalytic activity, in terms of the rate constant and the reaction half-time (t_1/2_), which decreased from 7.73·10^‑3^ min^−1^ to 4.71·10^−3^ min^−1^ and increased from 78 to 112 min, respectively.

The sample dialyzed for a long time (coll-dial_long-fd) had inferior properties in terms of porosity and crystallinity. Its XRD peaks were of very low-intensity (Figure 1c), which is probably due to increased disorder of the layers, or stacks of layers, but not degradation as suggested by the unchanged intensity of the IR band of D6R units (Figure 1e). The BET area dropped to 240 m^2^/g, but what is important, most of it constituted the external surface (175 m^2^/g); at the same time a significant drop of micropore volume (to 0.010 cm^3^/g) was not accompanied by an increase of the mesopore volume (0.02 cm^3^/g). For that reason, prolonged dialysis of the colloidal suspensions of zeolite layers is not recommended even if the acidity of the sample and catalytic activity were relatively good. The high acidity of the sample (BAS concentration 477 μmo/g, BAS_ext_ concentration 261 μmo/g) together with IR measurement in the pseudo-skeletal region (D6R vibration) suggest that amorphization of the sample is unlikely the cause for lower quality, and disorder and deformation of layers may be responsible for its observed XRD pattern with low-intensity peaks.

The MCM-56 zeolite in the powder form was also subjected to purification by dialysis (MCM-56 dial). After several days of treatment, the XRD pattern (Figure 1a) remained practically unchanged, as was the intensity of the D6R units in the IR spectra (Figure 1d), which suggests no important changes in crystallinity and layer disorder. Textural parameters were only slightly changed when compared to the material, which was calcined and then exchanged for NH_4_^+^ cations (MCM-56 exch); the change was close to the experimental accuracy of the method. Purification by dialysis did not change the HMI content (Figure 6a,b) but removed Na^+^ cations as efficiently as the combination of calcination followed by ion-exchange. The intensity of the IR maximum of pyridine adsorbed on Na^+^ in both cases was similar (Figure 6d). The calcined samples have higher BAS (669 vs. 405 μmol/g) and very similar LAS concentration (148 vs. 133 μmol/g); in both cases, the share of BAS_ext_ is also similar. The catalytic activities of both samples are also quite similar. The reaction half-time for reference, solid MCM-56 and the dialyzed sample is 65 and 79 min and reaction rates 9.11·10^−3^ min^−1^ and 8.20·10^−3^ min^−1^, respectively. Dialysis may be, therefore, recommended as an alternative method for purification of powder MCM-56 samples.

**Figure 7:**
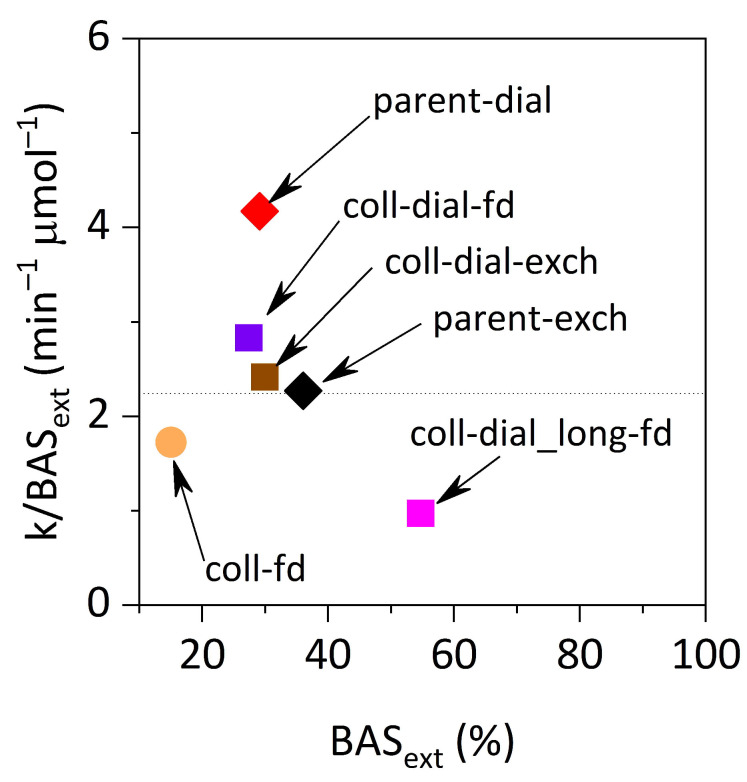
Dependence of the rate constant of Friedel–Crafts alkylation of mesitylene with benzyl alcohol normalized per concentration of the external Brønsted acid sites (k/BAS_ext_) on the fraction of external Brønsted acid sites (BAS_ext_). The dotted line marks the level for traditionally treated zeolite (calcination followed by ion exchange).

Acid strength is one of the important parameters influencing catalytic activity. Acid centers in hierarchical materials are very often weaker than in purely microporous ones, which may reduce or even completely reverse catalytic benefits of hierarchization, i.e., increasing accessibility [1]. Zhang et al. [27] compared catalytic performance of the series of materials with increased mesopore volume and proposed an interesting method of acid strength determination. They found that the Friedel–Crafts alkylation rate constant of mesitylene with benzyl alcohol normalized per concentration of the external Brønsted acid sites (k/BAS_ext_) varied only slightly, suggesting that the strength of BAS_ext_ was similar for all samples. The same results were obtained with the present MWW samples (Figure 7); the differences presented here are even smaller than in Zhang’s work. This shows that such extensive treatment as delamination/exfoliation with TBAOH, dialysis and subsequent flocculation by lyophilization did not change the acid strength of the external acid sites, which is the most important factor for conversion of bulky organic molecules. Colloidal dispersions of zeolite monolayers may be, therefore, attractive substrates to fabricate functional composite materials for catalysis.

## 3. Materials and Methods

*Synthesis*. The parent MCM-56 zeolite was synthesized using the published preparation procedure [22,33]. The following reagents were used: 50% NaOH solution, sodium aluminate (40–45% Na_2_O and 40–56% Al_2_O_3_), SiO_2_ nanoparticles (size 10–20 nm), 98% hexamethylene imine (HMI) solution, deionized water, in following molar ratios: 1 SiO_2_:0.04 Al_2_O_3_:0.093 Na_2_O: 0.3 HMI: 16H_2_O. All reagents, except for sodium aluminate (Riedel-de-Haen, Seelze, Germany), were purchased from Sigma-Aldrich Poland (Poznań, Polska). The crystallizations were carried out in a Teflon-lined autoclave (200 mL) with rotation at ambient temperature overnight and then at 143 °C for 38 h. The products were isolated by centrifugation, washed three times with distilled water and dried in air.

*Preparation of colloidal suspensions.* Colloidal suspensions were prepared by the reported two-step procedure [22]. In a typical preparation, 0.5 g of a MCM-56 sample, 7.50 g of 10–11.5% TBAOH and 18.50 g of H_2_O were mixed and stirred for 1.5 h and then centrifuged for 20 min at 10,000 rpm in a 50 mL Falcon tube using high-speed Z326 centrifuge (Hermle, Wehingen, Germany). After decantation, 40 mL of deionized water was added to the sediment, stirred for another 1.5 h and re-centrifuged under the above-mentioned conditions. The supernatant liquid was decanted and used as a stock solution with MWW layers for further processing and modification. Yields between 50 and 70% of the starting amount of the original MCM-56 solid are reached after precipitation; additional treatments do not result in further loss of the solid.

*Sample dialysis.* SpectaPor tubing with the cutoff of 12,000 Daltons was used (Avantor, Polska). As a standard, the samples were cleaned to pH in the range of 7–8, but in some cases also to the pH = 6–7.

*Freeze drying procedure.* Lyophilization (freeze-drying) was carried out using Labconco^®^ FreeZone and shell freezer (Kansas City, MO, USA). Samples were frozen for 20 min at −42 °C with rotation, which allowed obtaining spatially disoriented materials. The frozen samples were maintained under vacuum (<0.1 mbar) at −49 °C until the complete removal of H_2_O and volatile components.

*Catalyst characterization*. X-ray powder patterns were recorded using a Bruker AXS D8 Advance diffractometer (Bruker, Billerica, MA, USA) equipped with a graphite monochromator, position-sensitive detector (Våntec-1) using CuK_α_ radiation in Bragg–Brentano geometry in the range 1–10° 2θ and Rigaku MiniFlex diffractometer (Rigaku, The Woodlands, TX, USA) in refection mode, using CuK_α_ radiation (λ = 0.154 nm) in the ranges 3–30° 2θ. The XRD patterns were collected with steps of 0.02°.

The samples were examined under scanning electron microscopy (SEM) using a JEOL JSM5500LV scanning electron microscope (Tokyo, Japan). For the measurements, crystals were covered with a thin platinum layer by sputtering in the vacuum chamber of a BAL-TEC SCD-050 sample sputter coater (Los Angeles, CA, USA).

Infrared (IR) quantification of Lewis (LAS) and Brønsted (BAS) acid sites was made based on the adsorption of pyridine (Py), absorption coefficients: ε_LAS_ = 0.165 cm^2^/μmo, and ε_BAS_ = 0.044 cm^2^/μmo [34] and quantification of external Brønsted (BAS_ext_) acid sites by pivalonitrile (PN) [35] on the instrument Tensor 27 from Bruker (Ettlingen, Germany), MTC detector, spectral resolution 2 cm^−1^. Zeolites samples were pressed into self-supporting wafers and activated in situ at 475 °C for 1 h at a high vacuum (10^−5^ mbar) in a home-made quartz cell. The cell construction allowed in situ activation, measurement of the spectra at chosen temperature and adsorption of gases and vapors inside the infrared spectrometer. Before the adsorption of probe molecules, the system was cooled to the proper adsorption temperature: 170 °C for pyridine (Py) and ambient temperature for pivalonitrile (PN). After adsorption of the vapors (ca. 20 mbar equilibrium pressure), the gas phase together with weakly adsorbed species was desorbed at adsorption temperature for 20 min. To calculate the fraction of external Brønsted acid sites (BAS_ext_), the integral over the IR band characteristic of Si–OH–Al groups before and after PN adsorption was calculated, the former representing all BAS, the latter BAS inaccessible to PN, therefore, located at the external surfaces of the layers. All spectra were recalculated to the same pellet mass, equal 10 mg.

To record spectra in the KBr technique, 300 mg of KBr and ca. 3 mg of zeolite were mixed and pressed into a pellet with a 2 cm diameter. The intensity of spectra was normalized by calculating the integral over the 800 cm^−1^ maximum and normalizing it to unity (value of 1.0). Subsequently, the integral of the 560 cm^−1^ double maximum of the D6R unit was calculated.

Nitrogen isotherms were determined by the standard method at −196 °C (liquid nitrogen temperature) using an ASAP2025 (Micromeritics) static volumetric apparatus (Micromeritics, Norcross, GA, USA) and autosorb iQ gas sorption system (Quantachrome, Boynton Beach, FL, USA). Before experiments, the samples were outgassed at 350 °C using a turbomolecular pump to remove pre-adsorbed water.

*Catalysis.* The catalytic test reaction, liquid phase alkylation of mesitylene with benzyl alcohol, was carried out in a three-neck round-bottom flask equipped with a reflux condenser with heating in a multi-experiment workstation StarFish (Radleys Discovery Technologies, Saffron Walden, UK) under atmospheric pressure. The reaction temperature was 80 °C. Samples were activated at 500 °C for 5 h in air. Then, 19 g of mesitylene, 50 mg of the studied catalyst and 0.1 g of dodecane, as an internal standard, were mixed. The reaction mixture was maintained for 30 min at 80 °C, and then 0.2 g of benzyl alcohol was added. This was considered as the beginning of the reaction. Liquid samples (0.4 mL) were withdrawn at regular intervals and analyzed by the gas chromatograph PerkinElmer Clarus 600 GC (PerkinElmer, Shelton, CT, USA) with an FID detector (PerkinElmer, Shelton, CT, USA) using a 30 m packed column Elite-1MS (PerkinElmer, Shelton, CT, USA).

The alkylation reaction data were fitted to a pseudo-first-order rate law towards the limiting reactant (benzyl alcohol) in a linear region of 3–30 min of reaction to calculate apparent rate constants (k) and the half-time (t_1/2_) of reaction.

## 4. Conclusions

Isolation and reassembly of zeolite monolayers obtained by liquid exfoliation of the MCM-56 zeolite, with optional purification by dialysis or ammonium exchange, were investigated. The layers were recovered by flocculation with methanol and ammonium nitrate, and by freeze-drying. Flocculation alone, even with ammonium nitrate, did not ensure removal of residual sodium cations resulting in catalysts with low activity and showing that in the presence of a template, the Na^+^ for NH_4_^+^ exchange was ineffective. Dialysis, both of the colloids and the reference MCM-56 sample, proved to be efficient in removing sodium cations and preserving original layer microporosity. The colloids were also isolated by freeze-drying, producing very good quality samples with high BET area and micropore volume. They were also very fluffy and easy to handle. All dialyzed samples were active in the test reaction, Friedel–Crafts alkylation of mesitylene, with conversions much higher than MCM-22 (representing 3D zeolites) and comparable to MCM-56 (lamellar 2D zeolite). The applied treatments did not change the acid strength of the external acid sites, which are the most important for converting bulky organic molecules. It was proven that zeolite acidity was not degraded after dispersion into single monolayers by the reaction with tetrabutylammonium hydroxide and subsequent purification by dialysis and precipitation by freeze-drying, showing the potential of the colloid dispersion of zeolite layers in the formation of active catalysts. The yields of conversion of MCM-56 into monolayers were as high as 70%.

## Figures and Tables

**Table 1:** Textural (specific surface area S_BET_, external surface S_ext_, micropore V_micro_ and mesopore volumes V_meso_), acidic (concentration of Brønsted acid sites BAS, external Brønsted acid sites BAS_ext_, and Lewis acid sites LAS) parameters, and catalytic performance in terms of the reaction half-time (t_1/2_) and reaction constant (k) of zeolites under study.

Sample	Specific Surface Area, m^2^/g			Pore Volume, cm^3^/g			Acid Sites Concentration, μmol/g			Catalysis	
	S_BET_	S_ext_	S_ext_/S_BET_		V_micro_	V_meso_	BAS	LAS	BAS_ext_	t_1/2_, min	k·10^−3^, min^−1^
MCM-22	375	76	0.23		0.109	0.01	728	67	218	167	0.29
MCM-56	514	225	0.44		0.087	0.04	669	148	241	65	9.11
MCM-56 dial	539	242	0.45		0.085	0.05	405	133	118	74	8.20
coll-NH4	376	246	0.61		0.035	0.02	0	0	0	-	0.63
coll-EtOH	405	247	0.65		0.052	0.02	0	0	0	-	1.15
coll-fd	502	220	0.44		0.086	0.04	306	149	46	-	1.32
coll-dial-fd	566	171	0.30		0.108	0.08	598	180	164	79	7.73
coll-dial_long-fd	240	175	0.73		0.010	0.02	477	100	261	110	4.22
coll-dial-exch	438	51	0.12		0.070	0.19	391	79	117	112	4.71

## Data Availability

Data is contained within the article.

## Sample Availability

Not available.
